# Language interpretation in travel guidance platform: Text mining and sentiment analysis of TripAdvisor reviews

**DOI:** 10.3389/fpsyg.2022.1029945

**Published:** 2022-10-13

**Authors:** Miao Chu, Yi Chen, Lin Yang, Junfang Wang

**Affiliations:** ^1^Department of Marxism, Xi'an Jiaotong University, Xi'an, China; ^2^Department of Electronic Information, Hangzhou Dianzi University, Hangzhou, China; ^3^Department of Journalism and New Media, Xi'an Jiaotong University, Xi'an, China

**Keywords:** sentiment analysis, BERT, online reviews, electronic word of mouth, travel-related UGC, TripAdvisor

## Abstract

The opinions and feelings expressed by tourists in their reviews intuitively represent tourists' evaluation of travel destinations with distinct tones and strong emotions. Both consumers and product/service providers need help understanding and navigating the resulting information spaces, which are vast and dynamic. Traditional sentiment analysis is mostly based on statistics, which can analyze the sentiment of a large number of texts. However, it is difficult to classify the overall sentiment of a text, and the context-independent nature limits their representative power in a rich context, hurting performance in Natural Language Processing (NLP) tasks. This work proposes an aspect-based sentiment analysis model by extracting aspect-category and corresponding sentiment polarity from tourists' reviews, based on the Bidirectional Encoder Representation from Transformers (BERT) model. First, we design a text enhancement strategy which utilizes iterative translation across multiple languages, to generate a dataset of 4,000 reviews by extending a dataset of 2,000 online reviews on 1,000 tourist attractions. Then, the enhanced dataset is reorganized into 10 classifications by the Term Frequency-Inverse Document Frequency (TF-IDF) method. Finally, the aspect-based sentiment analysis is performed on the enhanced dataset, and the obtained sentiment polarity classification and prediction of the tourism review data make the expectations and appeals in tourists' language available. The experimental study generates generic and personalized recommendations for users based on the emotions in the language and helps merchants achieve more effective service and product upgrades.

## 1. Introduction

Word of mouth (WOM) plays a considerable role in influencing and forming consumers' attitudes and behavioral intentions (Sen and Lerman, 2007; Reza Jalilvand and Samiei, 2012a,b; Reza Jalilvand et al., 2012). Since online communication and virtual interactions have become commonplace, the importance of electronic word of mouth (eWOM) or online WOM is increasing. Online reviews as user-generated content (UGC) are a major part of eWOM (Ladhari and Michaud, 2015; Liu and Park, 2015; Banerjee and Chua, 2016; Anubha and Shome, 2020). Especially for intangible products and purchasing experiential goods (e.g., destinations, hotels, restaurants, and other tourism products), it is difficult to try out products before consumption. Therefore, tourists tend to rely heavily on online reviews to learn about the reputation of destinations or tourism facilities to make consumption decisions (Nguyen and Tong, 2022). According to Porteous (2014), nearly half of online consumers indicated that travel-related online reviews significantly influence their desire to visit a travel destination and they actively read and post reviews after experiencing service products. The eWOM of a tourist destination is largely equivalent to the consumption evaluation conveyed by the online reviews created by visitors to the destination. Therefore, this paper considers the online reviews of travel destinations to be eWOM.

The eWOM communication of tourist attractions is extremely significant, not only because it determines the consumption behavior of potential tourists (Zhu et al., 2015; Mohammed Abubakar, 2016; Jalilvand and Heidari, 2017) but, more importantly, it is a more trustworthy information source, which has a more powerful communication effect than tourism enterprises' propagation. This is partly because online reviews are less commercially motivated and allow for timely two-way information exchange, which gives potential visitors a stronger sense of natural trust. In addition, as UGC, online reviews often contain a lot of emotional expression, detailed descriptions, and intuitive feelings, which can make potential users have a more immersive, empathetic feelings. Therefore, eWOM has more influence on the consumption choices of tourists than the propagation of tourism enterprises (Reza Jalilvand et al., 2012).

Given the proliferation of reviews on online travel sites and the resulting consumer impact (TripAdvisor, 2019), many scholars have made efforts to explore the relationship between online travel reviews and consumer behavior, and to what extent reviews influence the consumer's decisions and choices (Hlee et al., 2018; Liu et al., 2019; Nguyen and Tong, 2022). Some studies tend to evaluate the content quality of online reviews. Among these, what makes a review “useful” is the universal central research question (Korfiatis et al., 2012; Li et al., 2021; Liu and Hu, 2021). Yin et al. (2014), and Kim and Hwang (2020) have proved that the emotion or tone conveyed in the text directly affects the usefulness of text communication or eWOM communication. However, measuring the sentiment conveyed in online review language, especially judging the polarity of visitors' emotions, is still in its infancy. Although there has been a long-term development in computer and other engineering disciplines to combine deep learning methods and textual information retrieval techniques for sentiment analysis (Zhao et al., 2017; Yiwen et al., 2022), its application in social sciences including tourism, linguistics, and communication is still scarce.

At present, sentiment analysis methods are mainly divided into two types: statistical-based and deep learning-based sentiment analysis methods. Statistical-based approaches determine the sentiment direction of an entire document based on certain words or phrases in the document (Birjali et al., 2021). Many lexicons are currently created manually, and manual approaches require human intervention to annotate the lexicon, a process that often requires significant time and labor costs (Taboada et al., 2011). In contrast, the deep learning-based sentiment analysis model is learned through neural networks, allowing the network model to predict the content of the next word based on contextual information without relying on an artificially labeled corpus. Deep learning-based methods can effectively solve the problem of ignoring contextual semantics in traditional sentiment analysis methods. The current typical neural network learning methods are convolutional neural network (CNN) (Wang et al., 2022), recurrent neural network (RNN) (Al-Smadi et al., 2018), long-short-term memory network (LSTM) (Priyadarshini and Cotton, 2021), and Transformer (Naseem et al., 2020), etc. The Bidirectional Encoder Representation from Transformers (BERT) (Devlin et al., 2019) is different from previous models in that it is a deep, bi-directional, unsupervised language representation model that can be resumed on top of the latest pre-trained context-sensitive language representation work. Compared with the comprehensiveness of BERT, CNN is recognized as having feature extraction locality and cannot extract global features of reviews; while RNN cannot be applied to long-term sequences. When the length of comments is too large, RNN will not be able to connect relevant information; LSTM cannot be used for parallel computing, which consumes a lot of time and space for experiments; Transformer lacks modeling of the time dimension, making the output of each position are very similar, which will eventually lead to poor performance in location classification. Considering these advantages of BERT, this paper choose BERT as the sentiment analysis model for this paper.

There have been some studies using various deep learning methods for sentiment analysis of reviews. Martín et al. (2018) used hotel-related reviews to carry out comparative experiments using CNN and LSTM to conduct sentiment analysis texts. Aljedaani et al. (2022) conducted sentiment analysis on online reviews of six US airlines, mainly using four dictionary-based and deep learning models including CNN, LSTM, etc. In addition, sentiment analysis of travel reviews has some research basis, but the research methods basically stay in the traditional techniques based on word filtering, co-occurrence analysis and semantic clustering (Ainin et al., 2020; Jardim and Mora, 2022). However, as far as we know, there are few research results on sentiment analysis of tourism reviews using the BERT model. Therefore, the use of BERT model, as a new means to explore the emotional state of tourists in tourism reviews, is an important innovation of this paper. In this paper, BERT language model is used to perform category recognition sentence-level sentiment classification (Gao et al., 2019) and sentiment analysis (Vaswani et al., 2017; Devlin et al., 2019) on the travel comments. The primary objective of this study is to answer the research questions of “what travel elements greatly affect tourists' emotions? What elements of travel motivate travelers to leave emotionally charged reviews on travel platforms?” This paper helps address the research gap and adds to sentiment analysis of online travel reviews through the application of the BERT model.

## 2. Data and methodology

**Figure 1:**
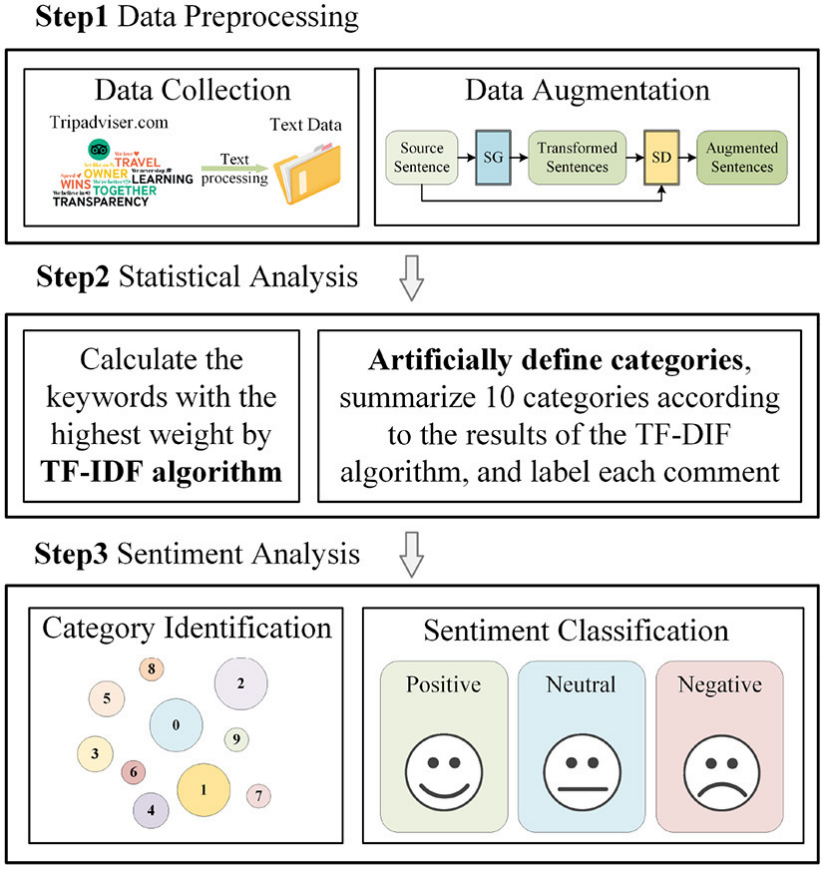
Sentiment analysis flowchart.

In present 10 comment categories about travel, the model performs sentiment analysis prediction for each comment and classifies them into Positive, Neutral, and Negative. The work in this paper can be divided into three steps, (1) data preprocessing, (2) statistical analysis, and (3) sentiment analysis. The specific schematic diagram is shown in Figure 1. The algorithmic process of Step 1, Step 2, and Step 3 will be described in detail.

### 2.1. Data preprocessing

This paper cites the dataset collected by Gurjar and Gupta (2021). This author first obtained a list of the top 1,000 attractions from lonelyplanet.com and subsequently obtained 2,000 reviews associated with them on tripadvisor.com based on the names of the attractions in this list. Because the training of neural networks needs sufficient data, we suggest that the small amount of data contained in this dataset may make the robustness of the results using this dataset poor. Therefore, in this paper, an iterative translation-based text enhancement method was used (Lee et al., 2021) to enrich the original dataset and expand the 2,000 reviews contained in the dataset to 4,000 reviews, so as to improve the reliability and generalization of the experiment. Existing rule-based text enhancement methods have a well-known limitation that they cannot be applied to other languages because the text depends on grammatical and structural features. Currently, Generative Adversarial Networks (GAN) (Qi et al., 2021) is one of the main research directions in text enhancement, however, GAN is less stable in training and relies on pre-training to predict the maximum likelihood. This paper proposes a new design for data enhancement method consisting of a sentence generator (SG) and a sentence discriminator (SD) based on GAN (Lee et al., 2021). The two are trained independently, with SG aiming to generate comprehensive enhanced utterances by serial and parallel iterations of existing translators, while SD is similar to a text classifier. Since SG cannot always generate high-quality transformed utterances, it needs to learn to filter out low-quality sentences by SD, and the two complement each other as a way to generate high-quality datasets.

**Figure 2:**
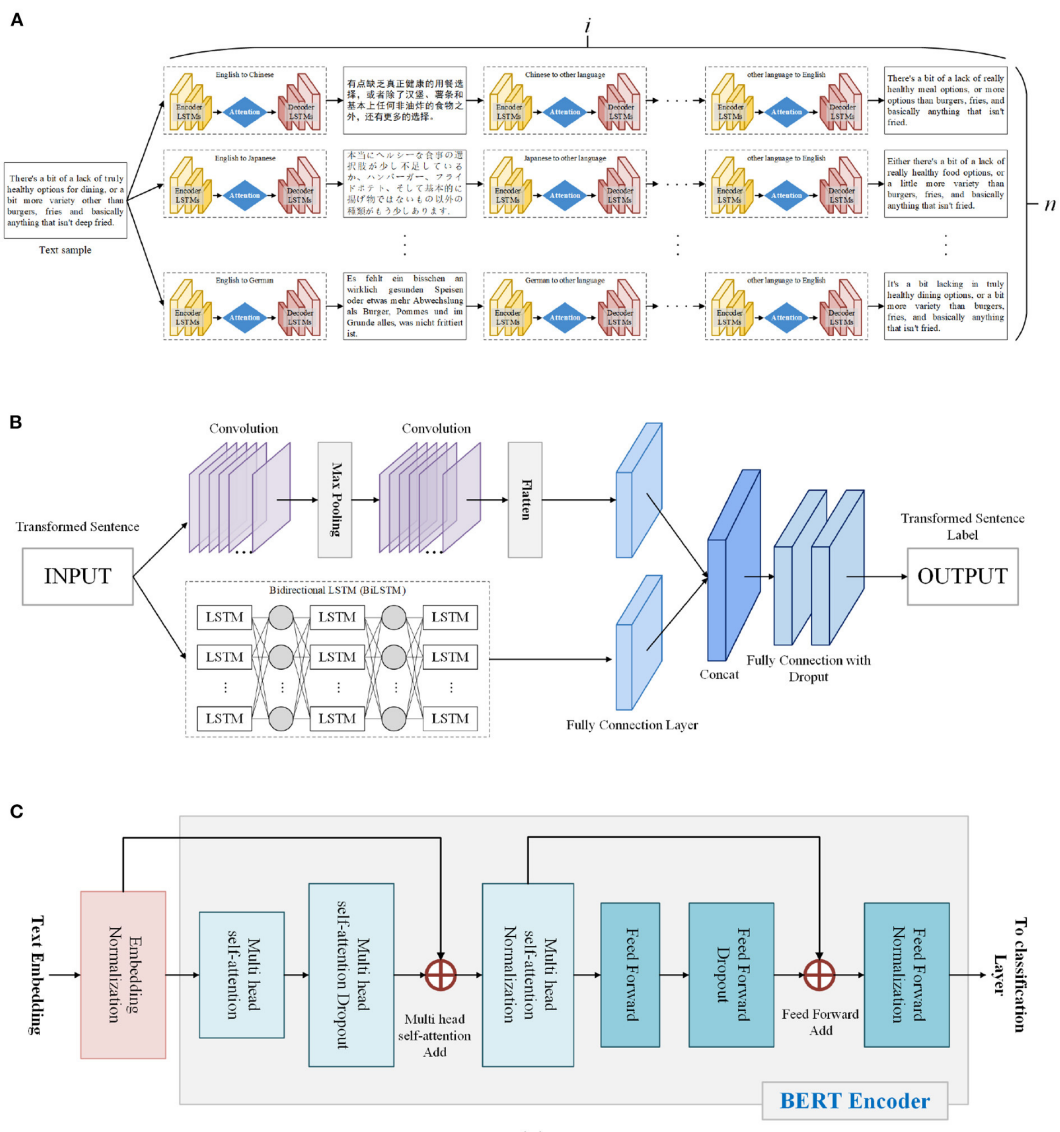
Structure of **(A)** SG, **(B)** SD, and **(C)** BERT.

The structure of SG is shown in Figure 2A. Sentence generator translates the source text into different languages by using Google Translate, which supports 109 languages, for *i* consecutive times, with the language randomly selected, and finally translates into the language of the source text. This work iterate the above operation in parallel for *n* times to obtain *n* converted sentences. This combination of serial and parallel translation methods can generate new utterances while preserving the grammatical and structural features of the text. A particularly crucial step in this algorithm is that if the converted sentence is identical to the source sentence, it is first deleted to prevent the generation of duplicate text. However, the percentage of duplicate sentences may still be high. This problem can be solved by adjusting *i*. The higher *i* will increase the conversion strength and successive text generators are less likely to generate duplicate sentences. However, high *i* values lead to trade-offs in generating sentences that are significantly different from the source sentences. If these over-translated sentences are used as training data, the performance of the model will degrade. Sentence discriminator will be used to solve the appealing problem, which is used to select and remove unnecessary transformed sentences for training.

The structure of SD is shown in Figure 2B. Sentence discriminator is based on a text data classifier that predicts the labels of SG-generated utterances after learning the source utterances. If the generated sentences do not match the source sentence labels, they are judged as misconverted sentences. The SD structure consists of a merged CNN and a parallel structure of BiLSTM (Liu and Guo, 2019), and the input of CNN-BiLSTM can be one or two sentences. When the input is one sentence, the input enters the CNN and BiLSTM layers in parallel and is concatenated by Concat; when the input is two sentences, the features of both are concatenated before Concat. Sentence discriminator will finally output the label classification of the sentences.

### 2.2. Keyword extraction and statistical analysis

In the statistical analysis step, this paper uses the Term Frequency-Inverse Document Frequency (TF-IDF) (Havrlant and Kreinovich, 2017) regression analysis to investigate the association between text data features and user travel plans. The TF-IDF (Havrlant and Kreinovich, 2017) is used as a weighting criterion to measure the word vector represented by the document to calculate the importance of terms in the text. His main idea is that if a word appears in a text with high TF frequency and rarely in other texts, the word or phrase is considered to have adequate representation and is suitable for classification. First, this paper uses *tfidf*_*i,j*_ as a parameter for text feature extraction, which indicates the frequency of word (keyword) occurrence in the text, and the calculation formula is as follows:

(1) $$\begin{array}{l} tf_{i,j}=\frac{n_{i,j}}{\sum_{k}n_{k,j}} \end{array}$$

where *tf*_*i,j*_ denotes the frequency of keywords in the dataset, *n*_*i,j*_ denotes the number of keywords in the dataset, and $\underset{k}{\sum }n_{k,j}$ denotes the sum of all words in the dataset.

Next, the inverse document frequency of keywords was calculated as follows:

(2) $$\begin{array}{l} idf_{i}=\log\frac{\left|D\right|}{|\left\{j:t_{i}\in d_{j}\right\}|+1} \end{array}$$

where |*D*| denotes the number of comments in the dataset and |{*j* : *t*_*i*_ ∈ *d*_*j*_}| indicates the number of frequencies containing keywords.

Finally, the TF-IDF of the keyword has been calculated, which is defined as follows:

(3) $$\begin{array}{l} F_{\text{freqi}}=tf_{i,j}\times idf_{i} \end{array}$$

### 2.3. Using BERT for sentiment analysis

The TF-IDF algorithm is used to calculate the frequency of the candidate product feature words, reflecting the importance of the feature words. After word frequency analysis of the dataset based on the TF-IDF algorithm, the word frequency weights generated by this dataset can be obtained. And based on this result, this paper summarizes 10 categories and labels each comment based on these 10 categories. Then, the BERT is used to classify the user sentiments in the dataset created in this paper. Google introduced BERT in 2018, which uses an attention mechanism to identify all other word-related contexts present in a text sequence. The bidirectional feature of BERT is used to learn the perspective of phrases centered on the surrounding environment. It consists of an encoder consisting of multiple self-attention modules with hidden layers. The model uses the previous and next contexts to generate the word representations present in the corpus. The structure is shown in Figure 2C.

Bidirectional Encoder Representation from Transformers is based on the transformer structure, but it only uses the encoder part of the transformer. Its overall framework is made up of multiple layers of transformer's encoder stacking. Existing BERT provides both simple and complex models. The simple model consists of 12 transformer blocks, of which the number of multi-head is 12. The complex model consists of 24 transformer blocks, of which the number of multi-head is 16. The main role of each attention is to recode the target word by its correlation with all the words in the sentence. The attention score is calculated in the self-attention layer using the product of input requests and keys, and then the attention score is normalized and multiplied with value using the softmax function. Finally, the association between words is calculated based on the weighted sum value. So, the calculation of each attention consists of three steps: (1) Calculating the correlation between words; (2) Normalizing the correlation; (3) Obtaining the encoding of the target word by weighting the sum of the correlation and the encoding of all words. Bidirectional Encoder Representation from Transformers contains two pre-training processes: Masked LM and Next Sentence Prediction (NSP). They are used to predict the sentiment from the transformed encoder output. Masked LM is used to solve the problem of unidirectionality in language models by randomly replacing the input words with [Mask] according to a certain percentage and then predicting these [Masks]. This paper swaps 15% of each phrase combination with an obscure word in the experimental part, and the classifier computes the actual phrase of the masked phrase based on the perception provided by the non-artifactual phrase in the sequence. During training, half of the next sentence is taken from the original comment, while the other half is taken from the dictionary. This two-way approach improves the efficiency of sentiment classification. The main task of NSP is to capture the relationships between sentences in the future. Sentence A and sentence B are randomly selected from the corpus at each training, 50% are correctly adjacent sentences and 50% are randomly selected sentences, and during training, the network determines whether sentence B is the next sentence of A.

Because BERT consists of Transformer, it needs to obtain pre-trained models on large datasets. For the sentiment classification problem, this paper uses pre-trained models trained based on BookCorpus (Zhu et al., 2015) and English Wikipedia. Based on the original network, two linear layers are used in the last layer to output the category probabilities with cross entropy as the loss, and after training on the dataset we built, the sentiment classification of travel reviews can be finished.

## 3. Results and discussion

### 3.1. General information

Through the TF-IDF algorithm, this paper calculates the weights of each keyword in the dataset and uses them as the categories for our subsequent sentiment analysis according to their high frequency of occurrence in the dataset. The categories are defined by selecting the keywords with the top 26 weights as our criteria for defining the categories, as shown in Table 1.

**Table 1:** Classification result.

**ID**	**Categories**	**Number**
0	Food {Delicious, unpalatable, spicy}	874
1	Price {Expensive, cheap, cost-effective}	655
2	Crowd {Congestion, queue, jammed}	507
3	Hygiene {Clean, dirty}	454
4	Couples {Dating, boyfriend}	360
5	Family {Parents, kids}	326
6	Age {Old, young, middle-aged}	308
7	Time {Morning, afternoon, night}	242
8	Handicap {Wheelchair, elevator, stairs}	193
9	Parking {Car, parking space}	81

As can be seen in Table 1, tourists' comments on tourism spots and facilities have obvious focus and emphasis. The travel review dataset used in this paper has more mentions of food, price, crowdedness, and sanitary conditions of locations. Because of the diversity of user comments, the related words are grouped into one broad category when counting. For example, the most frequent keyword in the data set is “food,” not that the word “food” appears too often in the data set, but this paper combines the words that describe food, such as it is not that the word “food” is too frequent in the dataset, but we will combine words that describe food, such as “delicious” and “unpalatable.” These words will be counted in the “Food” category, so our count is the sum of all words describing food in the dataset, with a total of 874 comments related to food. The second most frequent word is “Price,” with 655 comments on the level of local consumption. The third and fourth most frequent comments were about local transportation and hygiene, with 507 and 454 comments respectively. Among the ten categories mentioned above, visitors' comments on parking were the least frequent, with only 81 comments.

### 3.2. Different categories of sentiment analysis

Based on the above TF-IDF ten categories of comment data, this paper have experimented and tested the proposed dataset using the pre-trained BERT language model. This experiment evaluates two capabilities of the model, Aspect and Sentiment, respectively. For the former, the model is used to classify topics for unknown comments. The precision and recall of the BERT model on Aspect reached 92.68% and 89.42%, respectively. And for the latter, the model is used to predict sentiment for unknown comments. The precision and recall of the model on Sentiment reached 87.29% and 88.64%, respectively. The experiments prove that the BERT model is fully capable of the sentiment analysis task.

To illustrate the advantages of the data augmentation method used in this paper, the ablation experiments are conducts for the data augmentation method. Furthermore, experiments are executed on the sentiment analysis on the dataset without data augmentation and on the dataset with data augmentation. Experiment results show that the accuracy of the BERT model can reach 81.43% on the former and 87.29% on the latter. Compared with the dataset containing 2,000 reviews, the data augmentation method has been used to increase the accuracy of the BERT model by 5.86%, which fully demonstrates that the data augmentation method can improve the reliability of the model.

The required sentiment analysis results were also obtained in this paper. The results are shown in Table 2. Table 2 shows there are obvious differences in the polarity of each keyword. The relatively high-frequency categories (Food, Price, Crowd, Hygiene) in the dataset tend to be of negative polarity, the ratio of positive to negative values is generally small. The most frequent category “Food” in the dataset has only 90 ratings for “Positive,” and 683 ratings for “Negative.” The number of “Neutral” is similar to the number of “Positive,” which is 101. Of the 655 comments describing “Price,” the number of “Negative” comments was still more than five times greater than the number of “Positive” comments. The difference between “Negative” and “Positive” decreases as the number of reviews decreases. In the latter categories, the number of comments with the sentiment “Neutral” gradually dominates. Among the 81 reviews in the lowest frequency category of “Parking,” the number of “Positive” reviews exceeds that of “Negative” for the first time, with 68 reviews.

**Table 2:** Sentiment analysis results.

**Category**	**Food**	**Price**	**Crowd**	**Hygiene**	**Couples**	**Family**	**Age**	**Time**	**Handicap**	**Parking**
Total	874	655	507	454	360	326	308	242	193	81
Positive	90	78	95	113	84	80	52	48	27	68
Neutral	101	138	87	23	237	194	211	149	124	5
Negative	683	439	325	318	39	52	45	45	42	8
P/N	0.132	0.178	0.292	0.355	2.154	1.538	1.156	1.067	0.643	8.500

*P*/*N* represents the ratio of the number of *Positive* to the number of *Negative*.

Sentiment analysis shows that the desire of tourists to produce reviews is greatly influenced by their emotions. A large number of comments were written and published under the influence of negative emotions. With the decrease of creative enthusiasm, “Neutral” becomes the main emotional feature of critical text.

Table 2 also shows that tourists' negative evaluation of food, price, crowding, and hygiene is higher than other key factors, which represents the high expectation of tourists. Tourists are more likely to evaluate destination satisfaction through the above four factors and are more likely to generate user-produced content with WOM effects in the online environment because of these key factors. This is not only a catharsis of dissatisfaction, but also content producers believe that it can help subsequent tourists avoid risks to a certain extent, and also reduce the consumption desire of future tourists.

As an unintended result of this work, it appears that high satisfaction or generally positive comments about parking. However, the praise of parking does not mean that the 1,000 selected tourist attractions have a good performance in parking, but it is likely to indicate that tourists have relatively low parking conditions and requirements, and their expectations are easy to be met and leave praise.

## 4. Conclusion and implication

In this paper, the BERT network model is used for sentiment analysis to explore travel-related UGC on tourism platforms and the emotional polarity contained in the comment language. Bidirectional Encoder Representation from Transformers, as a new language representation model, has been well-applied in this study, showing great advantages in sentiment classification and sentiment rating prediction of text.

Our results show that tourists write more comments about food and hygiene on online platforms, and the expressions of these aspects are mostly negative emotions or tones. The comments on whether it is suitable for family and couples' trips were more agreeable, and the number of obviously negative or positive tone expressions in the comments were balanced. Comments about parking were the least frequent of all categories, but more than 83.95% of comments about parking were positive. This paper finds four destination variables that users care about the most and are prone to negative emotions, which lead to negative WOM: food, prices, crowding, and sanitation.

From these results, this paper responds to a long-standing question in the field of tourism and hotel management: what are a destination's core resources that tourists appreciate and care about (Bulchand-Gidumal et al., 2013). This study can derive some implications for hoteliers and managers of destination management organizations (DMOs), since this work empirically shows the extent of the relationship between the emotional expression of tourists in the review language and the eWOM of the tourist destination. Thus, DMO managers should focus on improving services in destinations, with particular emphasis on key elements that may contribute to negative WOM. Through this study, DMOs managers can find the deficiencies of destinations more accurately to improve their competitiveness more effectively.

## Data availability statement

The raw data supporting the conclusions of this article will be made available by the authors, without undue reservation.

## Author contributions

MC performed the theoretical analysis and wrote the first draft of the manuscript. YC was responsible for the data collection and analysis. LY contributed to conception and design of the study. JW contributed to the writing—review and editing. All authors contributed to manuscript revision, read, and approved the submitted version.

## Funding

This work was supported by National Social Science Fund of China (No. 17XKS029).

## Conflict of interest

The authors declare that the research was conducted in the absence of any commercial or financial relationships that could be construed as a potential conflict of interest.

## Publisher's note

All claims expressed in this article are solely those of the authors and do not necessarily represent those of their affiliated organizations, or those of the publisher, the editors and the reviewers. Any product that may be evaluated in this article, or claim that may be made by its manufacturer, is not guaranteed or endorsed by the publisher.
